# Is there a correlation between sensory impairments and social isolation in middle-aged and older Chinese population? Cross-sectional and longitudinal evidence from a nationally representative survey

**DOI:** 10.3389/fpubh.2023.1098109

**Published:** 2023-03-27

**Authors:** Ye Liu, Qinglei Sun, KaiweiSa Abuduxukuer, Yanan Hou, Jin Wei, Haiyun Liu, Jianfeng Luo, Guangfeng Gao, Yifan Zhou

**Affiliations:** ^1^Department of Ophthalmology, Putuo People's Hospital, Tongji University, Shanghai, China; ^2^Department of Biostatistics, School of Public Health, Fudan University, Shanghai, China; ^3^NHC Key Laboratory of Health Technology Assessment, Fudan University, Shanghai, China; ^4^Key Laboratory of Public Health Safety of Ministry of Education, Fudan University, Shanghai, China; ^5^Department of Ophthalmology, Shanghai East Hospital, Shanghai, China; ^6^Department of Endocrine and Metabolic Diseases, School of Medicine, Shanghai Institute of Endocrine and Metabolic Diseases, Ruijin Hospital, Shanghai Jiao Tong University, Shanghai, China; ^7^Department of Endocrine and Metabolic Diseases, School of Medicine, Shanghai General Hospital (Shanghai First People's Hospital), Shanghai Jiao Tong University, Shanghai, China; ^8^Department of Ophthalmology, School of Medicine, Shanghai General Hospital (Shanghai First People's Hospital), Shanghai Jiao Tong University, Shanghai, China; ^9^National Clinical Research Center for Eye Diseases, Shanghai, China; ^10^Shanghai Jiading Hospital of Traditional Chinese Medicine, Shanghai, China

**Keywords:** China Health and Retirement Longitudinal Study (CHARLS), sensory impairment, social isolation, social disconnectedness, loneliness

## Abstract

**Purpose:**

The aim of this study is to investigate the cross-sectional and longitudinal associations between sensory impairments (SIs) including single vision impairment (SVI), single hearing impairment (SHI), and dual sensory impairments (DSI) with social isolation in the middle-aged and older Chinese population.

**Methods:**

Data were obtained from the China Health and Retirement Longitudinal Survey (CHARLS). In total, 11,674 Chinese older adults aged over 45 were included at baseline 2011, and 6,859 participants who accomplished all four interviews from 2011 to 2018 were adapted for longitudinal analyses. Sensory status and social isolation measurements including social disconnectedness and self-perceived loneliness were collected. Assessment of social disconnectedness included the number of types of social activities in which they participated and the frequency of such participation. Loneliness referred to the subjective perception of loneliness. Other covariates included socio-demographic characteristics, medical conditions, and lifestyle-related factors. The impacts of baseline sensory status on social disconnectedness and loneliness were assessed using univariate and multivariate generalized linear models. A generalized linear model with generalized estimation equations (GEE) was used to assess the association between time-varying sensory statuses with social disconnectedness or loneliness over 8 years after being adjusted with multi-confounding factors.

**Results:**

Participants with SIs had significantly higher levels of social disconnectedness and self-perceived loneliness, compared to those who were free of SI. All kinds of SIs were significantly associated with loneliness according to both cross-sectional and longitudinal data. The correlations between DSI and social disconnectedness or loneliness at baseline and over 8 years were also noticed. SHI was found to be significantly associated with both frequency and types of social activities according to cross-sectional data and with the frequency of social activity participation in longitudinal analysis. SVI was only associated with the types of social activities at baseline (all *p-*values < 0.05).

**Conclusion:**

Sensory impairments, especially dual sensory impairments, have explicitly detrimental effects on social isolation among the older Chinese population. Over time, single hearing impairment specifically jeopardizes their frequency rather than types of social activities participation.

## 1. Introduction

A substantial increase in the size of the aging population in our society generates an urgent requirement for attaining a successful aging life, which fosters daunting challenges for biological, social, and medical science (1). Successful aging is multidimensional, which includes three main components: the lower probability of disease and related disability, maintenance of physical and cognitive function, and sustained engagement in social and productive activities (2).

In aging life, elderly people no longer need to work or work less, and they have more time for social engagement (3), which has become an important approach for older people to obtain social resources and preserve an active and healthy aging life. On the contrary, increasing pieces of evidence have also indicated that impoverished social relationships, also known as social isolation, have become a global public health challenge that influences health status (4), including cognitive impairment (5, 6) and depression (7), physical dysfunction or disability (8–11), multi-morbidities and mortality among the older population (9, 12, 13). Especially during the COVID-19 pandemic, as many countries have carried out various containment or lockdown policies which would inevitably lead to contrived social isolation, researchers have noticed that even temporary social disconnectedness could also impact health-related quality of life (HRQoL) (14), cognitive function (15, 16), and mental health status (16–18). Thus, to strengthen social relationships, encourage social participation, and reduce loneliness in an aging life, the investigation of modifiable risk factors of social isolation has gained certain attention as an important approach over the past decades (19).

Sensory impairments (SIs), including single hearing impairment (SHI), single vision impairment (SVI), and dual sensory impairments (DSI), which refer to the simultaneous presence of VI and HI, are commonly observed with aging (20). The associations of SIs with various adverse events in an aging life, including cognitive decline, physical dysfunctions, and all-cause mortality, have been widely investigated (21–23), which could also aggravate social isolation. Thus far, cross-sectional studies from some western countries have indicated that SI could restrict interpersonal communication and therefore inhibit social participation (24–26), while weaker associations or conflicting results were also noticed from other studies as well, which could be related to disparities in sample race, gender, aging categories, and adjustments of confounders (26, 27). To date, there are limited longitudinal reports on this issue, especially from developing countries.

China is the most populous developing country which is also facing a severe social problem of aging. Older Chinese are likely to neglect SI and related problems owing to their traditional attitudes regarding SI as a normal part of aging life, which might further contribute to the higher prevalence of SI and SI-related problems in China than that in some western countries (28). Several studies have indicated the associations between SI and adverse health consequences in the older Chinese population (29–31). However, to the best of our knowledge, there is a paucity of literature on SIs and social isolation in our aging population. To address the research gap, we conducted a cross-sectional and longitudinal observation on the associations between SIs and social isolation in a large, population-based sample from the China Health and Retirement Longitudinal Study (CHARLS). Allowing for the specific social institution, cultural background, and public health system in China, the purpose of this study is to verify whether SIs are independently associated with social isolation in the middle-aged and older population in China.

## 2. Methods

### 2.1. Participants and public involvement

Data were obtained from the China Health and Retirement Longitudinal Study (CHARLS), which is the first nationally representative longitudinal survey sampling residents (adults over 45 years old) from 450 villages/neighborhoods, and 150 counties across 28 provinces in China. With response rates over 80%, CHARLS provides the most up-to-date longitudinal data for the investigation of the health status and wellbeing of the middle-aged and elderly population in China. Initiated in 2011, CHARLS enrolled 17,708 participants at baseline (Wave 1), 18,254 participants at Wave 2 (2013), 20,273 participants at Wave 3 (2015), and 19,816 participants at Wave 4 (2018).

### 2.2. Measures

#### 2.2.1. Main outcome

Social isolation has been variously conceptualized. We quoted the definition of social isolation as “a state in which the individual lacks a sense of belonging socially, lacks engagement with others, has a minimal number of social contacts and they are deficient in fulfilling and quality relationships” according to Nicholson et al. (32). Such definition emphasized both subjective and objective dimensions of social isolation that are conceptualized as social disconnectedness and loneliness (33). Such quotation has been adapted in related studies (5, 34). Thus, the main outcome in this study falls in these two dimensions of social isolation according to the CHARLS questionnaire.

Social disconnectedness is an objective state reflecting a restricted social network and social inactivity with low participation and engagement. We focused on two aspects of social disconnectedness for quantification in the present study. First, Types: Respondents were asked whether they had participated in any of the following 11 activities in the past month including (1) Interact with friends; (2) Play Ma-jong, chess, cards, or go to community club; (3) Provide help to family, friends, or neighbors; (4) Go to a sport, social, or other kind of club; (5) Take part in a community organization; (6) Do voluntary or charity work; (7) Care for a sick or disabled adult; (8) Attend an educational or training course; (9) Stock investment; (10) Use the Internet; (11) Other activities. We summed the responses and created an index representing multifarious social participation, which revise-represented social disconnectedness, with higher scores reflecting lower levels of social disconnectedness (range: 0–11). Then, participants were asked, “How often do you usually do certain activities in the last month? Almost daily, almost every week, or not regularly?” We defined almost daily = 28 scores, almost every week = 8 scores, and not regularly = 3 scores, to assess the frequency of social participation. Scores of frequencies were also summed as one index for statistical analysis.

Loneliness is a subjective assessment defined as “a distressing feeling that accompanies the perception that one's social needs are not being met by the quantity or especially the quality of one's social relationships” according to Hawkley and Cacioppo (35). Respondents were asked the frequency of feeling lonely in the last week: 1. rarely or none of the time (< 1 day); 2. some or a little of the time (1–2 days); 3. occasionally or a moderate amount of the time (3–4 days); 4. most or all the time (5–7 days). Loneliness was dichotomized into 2 categories: 0, not lonely=those who reported feeling lonely rarely and none of the time, and 1, lonely=those who felt lonely sometimes, occasionally, or most of the time. This one-item measure correlates highly with multi-item loneliness scales and has been used in numerous studies (11, 34, 36).

### 2.3. Exposures

The main exposure in this present study is sensory status including no sensory impairment (NSI), single vision impairment (SVI), single hearing impairment (SHI), and dual sensory impairments (DSI). In CHARLS, VI consists of distal and near losses. Distal VI and near VI were evaluated by asking participants whether their eyesight was excellent, very good, good, fair, or poor when seeing things at a distance or up close, respectively. Reporting of fair or poor eyesight was classified as VI. Similarly, for the HI assessment, the question was: “Is your hearing excellent, very good, good, fair or poor.” A response of fair or poor hearing was identified as HI. Such assessment of SI has been widely used in previous CHARLS-related studies (29–31). DSI refers to the condition in which participants reported both VI and HI, and single SI refers to sole VI or HI without the other one.

Sensory impairment in the aging population could be amended by medical supports such as cataract surgery and hearing aids, or vice versa, get worse due to aging or pathologic progressions. Thus, along with baseline sensory status, we also investigated the impacts of time-varying SI statuses during 8 years of follow-up to further explore its longitudinal effects on social isolation.

### 2.4. Other variates

#### 2.4.1. Socio-demographic characteristics

Gender was a binary variable, male or female, and age was treated as a continuous variable. Marital status indicated whether the respondent lived alone or got accompanied. Participants who were separated, divorced, widowed, or never married were coded as “living alone,” while those who were married or partnered were coded as “living with partner.” Living area referred to urban or rural places where participants lived. Educational attainment represents one's social economic status, which was categorized into five groups: illiterate, less than elementary school, elementary school, middle school, and high school or above.

#### 2.4.2. Medical condition

Data on the physical condition were collected with the following question: “Have you ever been diagnosed by a doctor as having the following diseases: hypertension, dyslipidemia, diabetes, cancer, chronic lung diseases, liver diseases, heart disease, stroke, kidney diseases, memory-related diseases, digestive diseases, arthritis, and asthma?.” Suffering from more than two diseases was defined as multi-morbidities. Insurance covering referred to coverage of one or more kinds of health insurances, which represented an approach to health support.

#### 2.4.3. Lifestyle-related factors

The lifestyle variables included smoking and drinking status. Smoking was categorized as current/former smoker or never smoked. Drinking was a three-category variable that indicated the frequency of drinking: none, less than once a month, or more than once a month.

### 2.5. Statistical analysis

Statistical analyses were performed using SAS, version 9.4 (SAS Institute, Cary, NC, USA). In this study, the primary exposures of interest were SIs, while the other independent variables served as control variables. Baseline characteristics were compared among participants according to SI statuses (four groups) using the one-way ANOVA, Kruskal–Wallis test, or Chi-square test analysis based on obtained data. Generalized linear models were conducted to assess the associations between SIs, multiple covariates, and social isolation at baseline for cross-sectional analyses. Associations between time-variant SIs and social isolation measurements change over time (across four interviews over 8 years) were assessed using generalized linear models with generalized estimating equations (GEE), controlling for the intraindividual correlation between repeated measurements using an exchangeable correlation structure as previously described (37). To examine whether social isolation was dependent on the status of sensory impairments, models that adjusted for potential confounders as mentioned earlier were employed. Parameter estimates were shown with 95% confidence intervals and statistically significant was considered as a two-sided *p*-value of < 0.05. We begin our research with a hypothesis that a correlation exists between sensory impairments and social isolation, and the null hypothesis is the opposite stating that no such relationship exists.

## 3. Results

**Figure 1:**
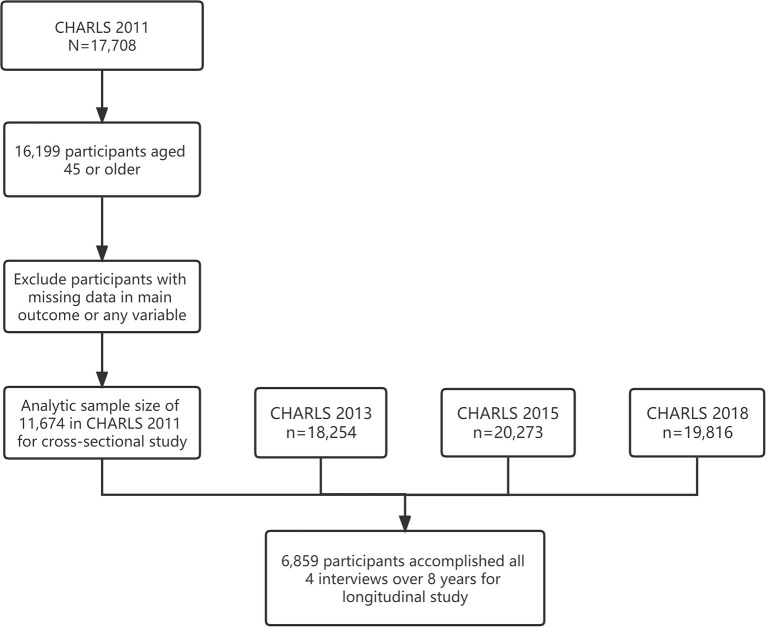
Graphic abstract of sample screening of the present study.

**Figure 2:**
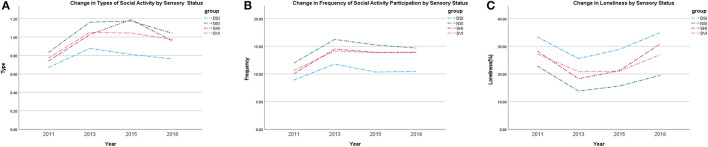
Changes of social disconnectedness and loneliness over time, 2011–2018. NSI, No sensory impairment; SVI, Single vision impairment; SHI, Single hearing impairment; DSI, Dual sensory impairments.

In total, 11,674 participants over 45 years old from baseline CHARLS 2011 were deemed eligible for the current study, among which, 6,859 participants accomplished all four interviews from 2011 to 2018 and were adopted in the longitudinal analyses (Figure 1). The baseline socio-demographic characteristics, medical conditions, lifestyle-related factors, and social participation measurements of the study sample were grouped by sensory statuses and shown in Table 1. Participants who were free from any kind of SI appeared to have explicitly more abundant social activities with higher frequency and lower levels of self-perception of loneliness at baseline. Variations in the number of activity types, frequency of social activities, and loneliness over 8 years are shown in Figure 2.

**Table 1:** Characteristics of participants in the present study from CHARLS 2011.

**Variables**	**Total**	**NSI**	**SHI**	**SVI**	**DSI**	***P*-value**
Age	59.38 ± 9.69	56.86 ± 8.78	59.69 ± 9.92	59.22 ± 9.27	61.44 ± 9.97	<0.0001
**Gender**						<0.0001
Male	5,572 (47.73)	1,892 (50.68)	1,037 (51.57)	687 (43.90)	1,956 (44.81)	
Female	6,102 (25.27)	1,841 (49.32)	974 (48.43)	878 (56.10)	2,409 (55.19)	
**Education**						<0.0001
Illiterate	3,121 (26.73)	800 (21.43)	504 (25.06)	445 (28.43)	1,372 (31.43)	
Less than elementary school	2,103 (18.01)	568 (15.22)	372 (18.50)	285 (18.21)	878 (20.11)	
Middle school	2,529 (21.66)	754 (20.20)	457 (22.73)	351 (22.43)	967 (22.15)	
High school or vocational school	2,423 (20.76)	924 (24.75)	423 (21.03)	299 (19.11)	777 (17.80)	
College and above	1,489 (12.83)	687 (18.40)	255 (12.68)	185 (11.82)	371 (24.77)	
**Marital status**						<0.0001
Living with partner	9,668 (82.82)	3178 (85.22)	1,658 (82.45)	1,310 (83.71)	3,522 (80.69)	
Living alone	2,006 (17.18)	555 (14.87)	353 (17.55)	255 (16.29)	843 (19.31)	
**Living area**						<0.0001
Urban	4,727 (40.49)	1,728 (46.29)	773 (38.44)	644 (41.15)	1,582 (36.24)	
Rural	6,947 (59.51)	2,005 (53.71)	1,238 (61.56)	921 (58.85)	2,783 (63.76)	
**Drinking status**						0.0005
Drink more than once a month	2,900 (24.84)	975 (26.12)	531 (26.40)	383 (24.47)	1,011 (23.16)	
Drink but less than once a month	904 (7.74)	323 (8.65)	157 (7.81)	100 (6.39)	324 (7.42)	
None of these	7,870 (67.41)	2,435 (65.23)	1,323 (65.79)	1,082 (69.14)	3,030 (69.42)	
**Smoking status**						0.0064
Yes	4,588 (39.30)	1,466 (39.27)	856 (42.57)	588 (37.57)	1,678 (38.44)	
No	7,086 (60.70)	2,267 (60.73)	1,155 (27.43)	977 (62.43)	2,687 (61.56)	
**Multi-morbidities**						<0.0001
Yes	2,203 (18.87)	450 (12.05)	377 (18.75)	284 (18.15)	1,092 (25.02)	
No	9,471 (81.13)	3,283 (87.95)	1,634 (81.25)	1,281 (81.85)	3,273 (74.98)	
**Insurance covering**						0.3582
Yes	10,918 (93.52)	3,498 (93.70)	1,888 (93.88)	1,448 (92.52)	4,084 (93.56)	
No	756 (6.48)	235 (6.30)	123 (6.12)	117 (7.48)	281 (6.44)	
**Social disconnectedness**						
Type	0.74 ± 0.93	0.85 ± 1.03	0.74 ± 0.92	0.76 ± 0.93	0.64 ± 0.82	<0.0001
Frequency	10.60 ± 16.35	12.50 ± 18.18	10.49 ± 15.83	11.09 ± 16.60	8.85 ± 14.57	<0.0001
**Loneliness**						<0.0001
Yes	3,456 (29.60)	875 (23.44)	560 (27.85)	455 (29.07)	1,566 (35.88)	
No	8,218 (70.40)	2,858 (76.56)	1,451 (72.15)	1,110 (70.93)	2,799 (64.12)	

The univariate generalized linear models indicated the potential associated factors of social isolation measurements in our sample at baseline 2011. SIs including SVI, SHI, and DSI, along with other factors including gender, age, educational level, living area, smoking, alcohol consumption, and medical conditions were all found to have certain associations with loneliness and the types or frequency of social activities (Table 2). Compared to single SI, DSI had lower incidence rate ratios and higher odds ratios, which suggests its potentially greater impacts on social disconnectedness and loneliness.

**Table 2:** Univariate generalized linear analysis of independent variables and social isolation measurements in 2011.

	**Social disconnectedness**				**Loneliness**	
**Variables**	**Type**		**Frequency**			
	**IRR (95%CI)**	***P*** **-value**	**IRR (95%CI)**	***P*** **-value**	**OR (95%CI)**	***P*** **-value**
**Gender**						
Male		Reference				
Female	0.878 (0.840, 0.919)	<0.0001	1.006 (0.927, 1.092)	0.8813	1.462 (1.349, 1.584)	<0.0001
**Age**	0.986 (0.983, 0.988)	<0.0001	0.997 (0.993, 1.002)	0.2255	1.202 (1.016, 1.024)	<0.0001
**Marital status**						
Live with partner		Reference				
Living alone	0.969 (0.913, 1.029)	0.3063	1.072 (0.962, 1.194)	0.2111	3.118 (2.825, 3.442)	<0.0001
**Education**						
Illiterate		Reference				
Less than primary school	1.206 (1.120, 1.300)	<0.0001	1.101 (0.973, 1.247)	0.1269	0.779 (0.694, 0875)	<0.0001
Junior School	1.352 (1.262, 1.449)	<0.0001	1.235 (1.098, 1.389)	0.0004	0.647 (0.578, 0.556)	<0.0001
Middle school or vocational school	1.597 (1.493, 1.708)	<0.0001	1.383 (1.229, 1.558)	<0.0001	0.494 (0.439, 0.556)	<0.0001
High school and above	2.362 (2.204, 2.531)	<0.0001	2.181 (1.910, 2.503)	<0.0001	0.370 (0.319, 0.429)	<0.0001
**Living area**						
Urban area		Reference				
Rural area	0.712 (0.681, 0.745)	<0.0001	0.626 (0.576, 0.680)	<0.0001	1.611 (1.482, 1.751)	<0.0001
**Smoke**						
No		Reference				
Yes	1.147 (1.096, 1.200)	<0.0001	1.031 (0.948, 1.121)	0.4817	0.842 (0.775, 0.914)	<0.0001
**Multi-morbidities**						
**Drinking status**						
No drink		Reference				
Drink but less than once a month	1.420 (1.315, 1.533)	<0.0001	1.239 (1.061, 1.446)	0.0068	0.852 (0.731, 0.993)	0.0402
Drink more than once a month	1.247 (1.186, 1.313)	<0.0001	1.097 (0.996, 1.207)	0.0594	0.748 (0.680, 0.824)	<0.0001
**Multi-morbidities**						
No						
Yes	0.979 (0.924, 1.038)	<0.478	0.971 (0.875, 1.078)	0.581	1.869 (1.697, 2.058)	<0.0001
**Insurance covering**						
No		Reference				
Yes	1.171 (0.824, 1.2900)	0.0015	1.144 (0.969, 1.352)	0.112	0.836 (0.715, 0.978)	0.0252
**Sensory status**						
No sensory impairment		Reference				
Hearing impairment	0.868 (0.813, 0.927)	<0.0001	0.839 (0.743, 0.948)	0.0048	1.260 (1.114, 1.426)	0.0002
Vision impairment	0.894 (0.832, 0.960)	0.0019	0.887 (0.777, 1.013)	0.0772	1.339 (1.172, 1.529)	<0.0001
Dual sensory impairment	0.754 (0.714, 0.796)	<0.0001	0.708 (0.642, 0.781)	<0.0001	1.827 (1.657, 2.015)	<0.0001

The results of the univariate generalized linear models indicated certain covariables that could probably confound the relationship between SIs and social isolation measurements in multivariate models. Thus, to further clarify the cross-sectional associations between SIs and social isolation measurements, we reanalyzed their relevance by controlling the covariates (Table 3). For the subjective perception of loneliness, all kinds of SIs showed profound and detrimental impacts after being adjusted for various confounders (all *p-*values < 0.001). DSI, also showed significant correlations with both the frequency (IRR: 0.779, 95% CI: 0.704–0.862; *p* < 0.001) and types (IRR: 0.861, 95% CI: 0.816–0.909; *p* < 0.001) of social activities. However, associations between the types of social activities and SHI or SVI were attenuated after being adjusted for lifestyle-related factors and medical conditions. Only SHI showed profound associations with the frequency of social activities after receiving an adjustment of socio-economic factors (Models 1 and 2; Table 3).

**Table 3:** Cross-sectional generalized linear analysis of sensory impairments and social isolation.

**Sensory status**	**Model 1** ^**a**^		**Model 2** ^**b**^		**Model 3** ^**c**^		**Model 4** ^**d**^	
	**IRR/OR**	**95%CI**	**IRR/OR**	**95%CI**	**IRR/OR**	**95%CI**	**IRR/OR**	**95%CI**
**Social disconnectedness: types**								
Ref: NSI^e^								
SHI^f^	0.868^***^	(0.813, 0.927)	0.898^**^	(0.841, 0.960)	0.940	(0.882, 1.002)	0.938	(0.879, 0.999)
SVI^g^	0.894^**^	(0.832, 0.960)	0.929^*^	(0.865, 0.997)	0.975	(0.910, 1.045)	0.975	(0.910, 1.045)
DSI^h^	0.754^***^	(0.714, 0.796)	0.805^***^	(0.762, 0.850)	0.864^***^	(0.819, 0.912)	0.861^***^	(0.816, 0.909)
**Social disconnectedness: frequency**								
**Ref: NSI**								
SHI	0.708^**^	(0.642, 0.781)	0.839^**^	(0.742, 0.948)	0.906	(0.802, 1.023)	0.907	(0.803, 1.024)
SVI	0.887	(0.777, 1.013)	0.885	(0.774, 1.011)	0.945	(0.810, 1.056)	0.926	(0.811, 1.057)
DSI	0.708^***^	(0.642, 0.781)	0.706^***^	(0.638, 0.780)	0.778^***^	(0.704, 0.860)	0.779^***^	(0.704, 0.862)
**Loneliness**								
**Ref: NSI**								
SHI	1.260^***^	(1.114, 1.426)	1.208^**^	(1.013, 1.022)	1.156^*^	(1.017, 1.314)	1.116	(0.981, 1.270)
SVI	1.339^***^	(1.172, 1.529)	1.256^**^	(1.098, 1.436)	1.234^**^	(1.076, 1.416)	1.196^*^	(1.042, 1.374)
DSI	1.827^***^	(1.657, 2.015)	1.663^***^	(1.505, 1.838)	1.593^***^	(1.437, 1.766)	1.495^***^	(1.347, 1.660)

The results of the generalized linear models were expressed as the incidence rate ratio (IRR) or odds ratio (OR) with 95% confidence intervals (CI). The analytic sample size was 11,674.

a Model 1: adjusted for demographic factors including age and gender;

b Model 2: adjusted for factors in Model 1, as well as social–economic including marital status, educational level, and living area;

c Model 3: adjusted for factors in Model 2, as well as lifestyle factors including smoking status and alcohol consumption;

d Model 4: adjusted for factors in Model 3, as well as medical conditions including multi-morbidities and insurance covering.

e NSI, No sensory impairment;

f SVI, Single vision impairment;

g SHI, Single hearing impairment;

h DSI, Dual sensory impairment.

* *p* < 0.05;

** *p* < 0.01;

*** *p* < 0.001.

In longitudinal analyses, associations between time-variant SIs and social isolation measurements over time were assessed using generalized linear models with generalized estimating equations, which could provide longitudinal observation of intraindividual correlations between repeated measurements. Consistent with cross-sectional analyses, we found that participants with DSI were more likely to involve in social activities with fewer types (IRR: 0.889, 95% CI: 0.855–0.925; *p* < 0.001) and less frequency (IRR: 0.825, 95% CI: 0.785–0.868; *p* < 0.001), and were more likely to experience loneliness (OR: 1.545, 95% CI: 1.418–1.684; *p* < 0.001) compared with participants without SIs after receiving adjustments of various confounders (Table 4). However, both single SI lost their associations with the types of social activities. Only SHI (IRR: 0.924, 95% CI: 0.859–0.994; *p* < 0.05) remained to be significantly associated with the frequency of social activities after being adjusted for covariates. We failed to find any profound correlation between SVI and the frequency of social activities in any of the four models.

**Table 4:** Longitudinal generalized linear analysis of sensory impairments and social isolation, 2011–2018.

**Sensory status**	**Model 1** ^**a**^		**Model 2** ^**b**^		**Model 3** ^**c**^		**Model 4** ^**d**^	
	**IRR/OR**	**95%CI**	**IRR/OR**	**95%CI**	**IRR/OR**	**95%CI**	**IRR/OR**	**95%CI**
**Social disconnectedness: types**								
**Ref: NSI** ^e^								
SHI^f^	0.955	(0.903, 1.011)	0.968	(0.915, 1.024)	0.977	(0.924, 1.032)	0.976	(0.924, 1.032)
SVI^g^	0.985	(0.942, 1.029)	0.992	(0.950, 1.036)	0.998	(0.957, 1.041)	0.997	(0.956, 1.040)
DSI^h^	0.854^***^	(0.820, 0.890)	0.869^***^	(0.835, 0.905)	0.891^***^	(0.857, 0.927)	0.889^***^	(0.855, 0.925)
**Social disconnectedness: frequency**								
**Ref: NSI**								
SHI	0.891^**^	(0.830, 0.956)	0.895^**^	(0.834, 0.962)	0.925^*^	(0.860, 0.995)	0.924^*^	(0.859, 0.994)
SVI	0.959	(0.909, 1.011)	0.956	(0.907, 1.008)	0.974	(0.923, 1.028)	0.973	(0.923, 1.027)
DSI	0.792^***^	(0.753, 0.833)	0.793^***^	(0.754, 0.834)	0.827^***^	(0.786, 0.870)	0.825^***^	(0.785, 0.868)
**Loneliness**								
**Ref: NSI**								
SHI	1.328^***^	(1.186, 1.486)	1.316^***^	(1.174, 1.475)	1.310^***^	(1.166, 1.473)	1.294^***^	(1.150, 1.456)
SVI	1.267^***^	(1.158, 1.387)	1.241^***^	(1.133, 1.360)	1.237^***^	(1.126, 1.359)	1.228^***^	(1.117, 1.350)
DSI	1.623^***^	(1.495, 1.763)	1.579^***^	(1.453, 1.716)	1.576^***^	(1.447, 1.716)	1.545^***^	(1.418, 1.684)

The results of the longitudinal generalized linear models were expressed as the incidence rate ratio (IRR) or odds ratio (OR) with 95% confidence intervals (CI). The analytic sample size was 6,859.

a Model 1: adjusted for demographic factors including age and gender;

b Model 2: adjusted for factors in Model 1, as well as social–economic including marital status, educational level, and living area;

c Model 3: adjusted for factors in Model 2, as well as lifestyle factors including smoking status and alcohol consumption;

d Model 4: adjusted for factors in Model 3, as well as medical conditions including multi-morbidities and insurance covering.

e NSI, No sensory impairment;

f SVI, Single vision impairment;

g SHI, Single hearing impairment;

h DSI, Dual sensory impairment.

* *p* < 0.05;

** *p* < 0.01;

*** *p* < 0.001.

## 4. Discussion

To the best of our knowledge, the present study is the very first study that provides explicit evidence of the detrimental impacts of sensory impairments on social isolation among a middle-aged and older Chinese population, using cross-sectional and longitudinal data from a nationally representative survey in China. The importance of social engagement to a healthy aging life has been highlighted as a global consensus (38). Thus, it is essential to investigate factors that may jeopardize social engagement, which consequently leads to social isolation and adverse events in aging life.

### 4.1. SIs and jeopardized social connection

Scales of sensory assessments, covariate adjustments, and evaluation of social isolation measurements including social disconnectedness and loneliness from previous study designs were widely divergent, which may lead to nuanced results and difficulties in direct comparisons among these studies (24, 26, 27, 39–42). Nevertheless, most findings still converged to a general and reasonable conclusion, that SIs and the consequent difficulties in communication could exert certain detrimental impacts on social participation to some extent (43). Results from both cross-sectional analyses and longitudinal observation in the present study reiterated that SIs, especially DSI, had explicit associations with higher social disconnectedness and increased loneliness among elderly Chinese. Our work echoed related cross-sectional studies in other countries (24, 26, 27, 39, 40), which also provided empirical longitudinal pieces of evidence on this issue in the Chinese population for the first time.

The complex mechanisms underlying “sensory-social isolation” correlation or interactions are yet to be explored. Along with direct obstructions to social interactions, older adults with sensory loss are more prone to functional (44) and mental health dysfunctions such as cognitive decline (44), depression, and anxiety (45), which may consequently jeopardize social communication. On the other hand, people experiencing sensory deprivation, especially over extended periods of time, often report perceptual disturbances such as visual and auditory hallucinations, which might be considered as a protective reaction of the social brain (46, 47). Even short-term sensory deprivation could induce psychotomimetic effects or cognitive appraisal distortion (48, 49). Such perceptual disorders propel them to become suspicious of interpersonal interaction and misinterpret social clues (46, 47).

### 4.2. DSI, combined effects, and compounded problems

Compared to single SI, DSI had more consistent and profound effects on social disconnectedness and loneliness according to both cross-sectional and longitudinal data in the present study. Links between SIs and social isolation are primarily mediated by communication difficulties (50). For people with single SIs, those who have SHI might compensate their inter-person communication using lip reading or sign language, while those who suffer from SVI can rely on spoken language. However, the combination of concurrent sensory deficits creates a compounded problem that restrains such cross-modal compensation (51, 52). Therefore, older people with DSI would more likely to be timid, hesitant, or confused, which inevitably leads to isolation, disappointment, and frustration during communication (24). Compared to people with single SI, those with DSI may appear to have a higher likelihood of social inactivity (27), and poorer scores in psychosocial variables and life quality evaluation (53). Also presumably, their communication partners might become impatient in an unjustified way, or pay inadequate attention in order to understand them (54). Thus, our findings underscore the importance of primary prevention and rehabilitation in both vision and hearing impairments as well as setting objectives to increase social participation and life satisfaction among people with sensory disabilities and the removal of environmental barriers (24).

### 4.3. Single SI jeopardizes the frequency, rather than the types of social activities

It engrossed our attention that, although cross-sectionally, single SI, especially SHI, could have certain impacts on both the frequency and types of social activities. However, during longitudinal observation, their impacts on types of social activities vanished and only SHI remained significantly associated with the frequency of social activities. We supposed that participation in specific types of social activities shall be regarded as very personal habits and customed lifestyles in the form of daily routine, which could have been preserved for a long time in one's aging life. Such personal habits might maintain to a certain extent through compensating for the use of another intact sensory organ as mentioned earlier during aging life with single SI. Such an explanation could partly account for the attenuated significance of the association between single SI and social disconnectedness, after being adjusted for lifestyle-related factors including smoking and alcohol consumption (Model 3; Table 3). Thus, we consider that the types of social activities in which one regularly takes part in could reflect his or her personal habits and customed lifestyle, which would not be so easily affected by single SI. On the contrary, sensory deficits, especially hearing impairment, might curtail the frequency of social activities by compromising physical functions, mobility, mental status, and the ability to communicate (29, 30). Hence, for the first time, our study may highlight that it is the frequency, rather than the types of social activities, that receive detrimental impacts from SI, especially hearing deficits over time in aging life. Such findings deserve certain attention and further research in the future.

### 4.4. SHI may exert more explicit impacts than SVI

As for single SIs, some researchers pointed out that VI seems to have more profound impacts than HI on social participation (24, 26), which they supposed might be explained that VI would exert more profoundly detrimental impacts on functional activity (55), mobility and social interactions (26). However, according to our cross-sectional and longitudinal data, we found that SHI may exert more explicitly negative impacts than SVI on the frequency of social activities among older Chinese over time (Tables 3, 4). One possible explanation for such a situation might lay in the fact that there is less feasibility and probability in the amelioration of hearing impairment than the visual deficit over time among aging Chinese. Assistive devices such as glasses and portable magnifiers are relatively efficient, economic, and easy-to-carry interventions for quite a lot of people who suffer reversible VI (56). On the contrary, interventions like wearing hearing aids for improvement of hearing status would more likely confront financial constraints, unfamiliarity with hearing aids, and difficulties during manipulating, which might all contribute to the fact that the application level of hearing aids is far less than expected among Chinese population (57). In addition to amplifying desired sounds, hearing aids would amplify noises as well, thus making users feel too loud and noisy. Such a muffled effect also jeopardizes peoples' belief in hearing aids (57). Some authors argued that, even when listening aids are used, the promotion of satisfaction might be limited in people with hearing deficits (58, 59). Therefore, untreated HI and the subsequent sustaining hearing deficit might exert more explicitly longitudinal effects on SP among our population over time.

### 4.5. Limitations and strengths

We need to acknowledge some limitations. Self-reports provide information about subjective perception and, therefore, are clinically highly relevant and suitable for studies on social participation and subjective perception of loneliness like ours (27). Questionnaires have also been considered as effective strategies for the early identification of sensory problems (24). However, we still need to notice the potential bias owing to the nature of self-reported data. Previous related studies adopted various definitions and evaluation methods of social isolation, which renders direct comparisons of the results from our study and those studies to be difficult. During the COVID-19 pandemic, many countries have carried out kinds of containment or restriction policies, which is different from the definition of social isolation in aging life from the current study. However, our study could still provide a meaningful reference for such social disconnectedness and call for even more attention to an aging population with sensory impairments. Interactions among social connections and physical and mental dysfunctions in an older population with sensory impairments deserve further exploration.

Nevertheless, our study is one of the very first studies to verify both cross-sectional and longitudinal associations between sensory impairments and social isolation. We obtained data from the first nationally representative survey on the health and wellbeing status of the middle-aged and elderly population in China (CHARLS) to provide explicit evidence on this topic. Findings from the current study could be generalized to the entire country or could be even used as a reference to other developing countries. Multiple associated factors were included and adjusted in this study analyses, which could otherwise potentially confound the relationship between sensory impairments and social isolation.

## 5. Conclusion

Sensory impairments, especially dual sensory impairments, have explicitly detrimental effects on social disconnectedness and loneliness among older Chinese. Single hearing impairment specifically jeopardized their frequency rather than types of social activities over time. Future studies are needed to determine the mechanisms underlying these associations.

## Data availability statement

Publicly available datasets were analyzed in this study. This data can be found here: The original dataset of CHARLS is accessible on http://charls.pku.edu.cn/.

## Ethics statement

The studies involving human participants were reviewed and approved by Institutional Review Board at Peking University. The patients/participants provided their written informed consent to participate in this study. Written informed consent was obtained from the individual (s) for the publication of any potentially identifiable images or data included in this article.

## Author contributions

YZ and JL: conceptualization. JW and KA: investigation and methodology. QS and YL: data curation, formal analysis, and writing—original draft. HL, YH, and GG: writing—review and editing and manuscript revision. All authors read and approved the final manuscript.
